# Oleuropein Supplementation Ameliorates Long-Course Diabetic Nephropathy and Diabetic Cardiomyopathy Induced by Advanced Stage of Type 2 Diabetes in *db*/*db* Mice

**DOI:** 10.3390/nu16060848

**Published:** 2024-03-15

**Authors:** Shujuan Zheng, Ruixuan Geng, Jingya Guo, Seong-Gook Kang, Kunlun Huang, Tao Tong

**Affiliations:** 1 Key Laboratory of Precision Nutrition and Food Quality, Key Laboratory of Functional Dairy, Ministry of Education, College of Food Science and Nutritional Engineering, China Agricultural University, Beijing 100083, China; zsj@ahpu.edu.cn (S.Z.); 17768128861@163.com (R.G.); guojy2017@126.com (J.G.); 2 College of Biological and Food Engineering, Anhui Polytechnic University, Wuhu 241000, China; 3 Department of Food Engineering, Solar Salt Research Center, Mokpo National University, Muangun 58554, Republic of Korea; sgkang@mokpo.ac.kr; 4 Key Laboratory of Safety Assessment of Genetically Modified Organism (Food Safety), Ministry of Agriculture, Beijing 100083, China; 5 Beijing Laboratory for Food Quality and Safety, Beijing 100083, China

**Keywords:** oleuropein, long-course diabetic nephropathy, long-course diabetic cardiomyopathy, transcriptomics

## Abstract

Previous studies have reported the therapeutic effects of oleuropein (OP) consumption on the early stage of diabetic nephropathy and diabetic cardiomyopathy. However, the efficacy of OP on the long-course of these diabetes complications has not been investigated. Therefore, in this study, to investigate the relieving effects of OP intake on these diseases, and to explore the underlying mechanisms, *db*/*db* mice (17-week-old) were orally administrated with OP (200 mg/kg bodyweight) for 15 weeks. We found that OP reduced expansion of the glomerular mesangial matrix, renal inflammation, renal fibrosis, and renal apoptosis. Meanwhile, OP treatment exerted cardiac anti-fibrotic, anti-inflammatory, and anti-apoptosis effects. Notably, transcriptomic and bioinformatic analyses indicated 290 and 267 differentially expressed genes in the kidney and heart replying to OP treatment, respectively. For long-course diabetic nephropathy, OP supplementation significantly upregulated the cyclic guanosine monophosphate-dependent protein kinase (cGMP–PKG) signaling pathway. For long-course diabetic cardiomyopathy, p53 and cellular senescence signaling pathways were significantly downregulated in response to OP supplementation. Furthermore, OP treatment could significantly upregulate the transcriptional expression of the ATPase Na^+^/K^+^ transporting subunit alpha 3, which was enriched in the cGMP–PKG signaling pathway. In contrast, OP treatment could significantly downregulate the transcriptional expressions of cyclin-dependent kinase 1, G two S phase expressed protein 1, and cyclin B2, which were enriched in p53 and cellular senescence signal pathways; these genes were confirmed by qPCR validation. Overall, our findings demonstrate that OP ameliorated long-course diabetic nephropathy and cardiomyopathy in *db*/*db* mice and highlight the potential benefits of OP as a functional dietary supplement in diabetes complications treatment.

## 1. Introduction

Long-term hyperglycemia can cause pathological changes in various organs and tissues, resulting in diabetes complications such as diabetic cardiomyopathy, diabetic nephropathy, diabetic neuropathy, and diabetic retinopathy [1,2]. The latest data (2021) from International Diabetes Federation showed that there were 537 million (20–79 years) diabetics worldwide, accounting for 10.5% of the adult population; by 2030, the number of diabetics is projected to be up to 643 million [3,4,5]. Clinical data show that approximately 30% to 40% of diabetics will develop diabetic nephropathy [6]. The morbidity of diastolic dysfunction in diabetics has been estimated to be about 52~60% [7]. In addition to harming health, diabetic nephropathy and diabetic cardiomyopathy also bring heavy financial burden to patients. Therefore, effective strategies are necessary to relieve these diabetes complications.

Oleuropein (OP), a phenolic compound which mainly presents in olive leaves, immature olive fruits, and extra virgin olive oil, exerts multiple biological functions [8,9]. OP has been shown to have beneficial effects on the early onset of diabetic nephropathy and diabetic cardiomyopathy [10,11,12]. However, the mitigating effects of OP on long-course diabetic nephropathy and diabetic cardiomyopathy have not yet been explored. Our earlier research demonstrated that OP alleviated the advanced stage of type 2 diabetes in *db*/*db* mice [13]. In detail, OP (200 mg/kg bodyweight) had been orally administered to 17-week-old *db*/*db* mice daily for 15 weeks. We found that OP significantly reduced the fasting blood glucose (339.5 mg/dL vs. 512.5 mg/dL) and the homeostasis model assessment–insulin resistance index (30.4 vs. 17.2), restored the histological characteristics of pancreatic islets and liver, and significantly upregulated the phosphorylation level of protein kinase B in the liver of *db*/*db* mice. According to our prior discoveries, we posited that supplementing with OP could effectively mitigate the long-course diabetic nephropathy and diabetic cardiomyopathy in *db*/*db* mice with advanced stage type 2 diabetes.

## 2. Materials and Methods

### 2.1. Materials

OP (analytical reagent grade) was procured from Chengdu Purify Technology Development Co., Ltd. (Chengdu, China). Chemical reagents of analytical grade were purchased from Macklin Chemical Reagent Co., Ltd. (Shanghai, China).

### 2.2. Animals and Experimental Design

**Figure 1:**
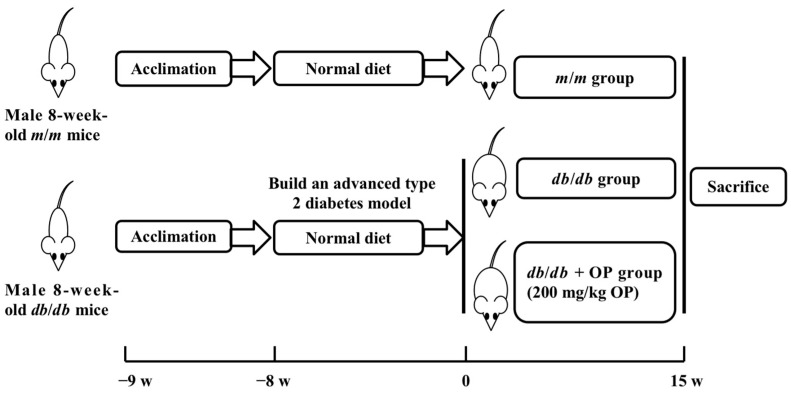
Schematic diagram of experimental design.

The animal experiment was approved by the Animal Ethics Committee of China Agricultural University (AW11099102-3-4, Beijing, China). Male BKS–*Lepr^em2Cd479^*/Gpt (*db*/*db*) mice (8-week-old) and age-matched wild-type control male BKS–DB (*m*/*m*) mice (SCXK (Su) 2018-0008) were purchased from Nanjing Collective Pharmachem Co., Ltd. (Nanjing, China). Mice were housed 3–4 per cage in a 12 h/12 h self-controlled daylight control system (lights on at 6:30 a.m.) in a specific pathogen-free animal room (SYXK (Jing) 2020-0052). The room temperature was 21 ± 2 °C, humidity was 40–70%, and air exchange frequency was 10~12 times/h. Studies have indicated that *db*/*db* mice at 16 weeks of age exhibit elevated casual blood glucose levels as well as high fasting blood glucose levels, making them a suitable model for studying the advanced stage of type 2 diabetes [14,15]. All animals were given free access to water and standard chow diet (Huafukang Biotechnology Co., Ltd., Beijing, China) until they reached 17 weeks of age. Then, the mice were divided into 3 groups: (1) *db*/*db* group (*n* = 7); (2) *db*/*db* + OP group (*n* = 8); (3) *m*/*m* group (*n* = 7). Mice in the *db*/*db* + OP group were administered OP aqueous solution via gavage at a dose of 200 mg/kg body weight daily for 15 weeks (Figure 1). The animals used in this study were the same as those in the research of Zheng et al. [13].

### 2.3. Sample Collection

After 15 weeks of treatment, the animals were fasted for 6 h and blood samples were collected from the posterior orbital venous plexus. Then, the mice were dissected. Part of the removed kidney and heart tissues were fixed with 4% paraformaldehyde solution, and the remaining tissue samples were snap-frozen in liquid nitrogen and subsequently stored at −80 °C.

### 2.4. Periodic Acid–Schiff (PAS) Staining

The fixed tissues were dehydrated using a gradient of ethanol, cleared in xylene, sealed in wax, and then sectioned into 4.5 µm slices. The sections were de-waxed and sequentially stained in periodate stain, chevron stain, and hematoxylin stain, followed by dehydration and sealing of the sections. Histopathological changes were observed under bright field using a Leica DM750 microscope (Leica, Nussloch, Germany).

### 2.5. Masson Staining

The fixed tissues underwent dehydration with a series of ethanol gradients, followed by clearing in xylene, wax sealing, and subsequent cutting into 4.5 µm sections. The sections were de-waxed and stained sequentially in potassium dichromate, ferric hematoxylin, ponpceau acid fuchsin, phosphomolybdic acid, and aniline blue. The sections were rinsed with 1% glacial acetic acid, dehydrated with anhydrous ethanol, and sealed with neutral gum.

### 2.6. Immunohistochemical Staining

The fixed tissues were dehydrated through a series of ethanol gradients, cleared in xylene, sealed in wax, and then sectioned into 4.5 µm slices. Sections were de-waxed and antigen repair was performed. Sections were then blocked with serum, incubated sequentially with primary and secondary antibodies, chromogenized with diaminobenzidine (DAB), re-stained with nuclei, and finally sealed.

### 2.7. Terminal Deoxynucleotidyl Transferase (TdT) dUTP Nick End Labeling (TUNEL) Staining

The fixed tissues were dehydrated through a series of ethanol gradients, cleared in xylene, sealed in wax, and then sectioned into 4.5 µm slices. After paraffin removal, antigen repair was performed on the slices, followed by TUNEL reaction, DAB staining, and finally sealing.

### 2.8. Real-Time Quantitative PCR

Total RNA was extracted from the tissues using TRIzol^TM^ reagent (TIANGEN, Beijing, China) [13]. A cDNA Synthesis SuperMix (TransGen Biotech, Beijing, China) kit was used to synthesize the cDNA. Then, a SuperReal PreMix Plus (SYBR Green) (TransGen Biotech, Beijing, China) kit was used to perform real-time quantitative PCR. Primer sequences for qPCR are in Table 1. The real-time PCR quantitative amplifications were performed as follows: 95 °C for 3 min, 40 cycles of 95 °C for 10 s, and 55 °C for 30 s. The expression of β-actin was utilized as a reference to normalize mRNA expression levels.

### 2.9. Transcriptome Sequencing

After extracting tissue RNA, genomic DNA was removed by using DNase I (TransGen Biotech, Beijing, China). Quality inspection was conducted on the obtained RNA to meet the RNA library construction and sequencing requirements. The TruSeqTM RNA sample preparation kit was used to establish an RNA library. Then, the mRNA was enriched and braked. After amplification of cDNA using the mRNA as a template by PCR, cDNA fragments of 200~300 bp in size were screened by DNA Clean Beads. The prepared library was sequenced on an Illumina NovaSeq 6000 sequencing platform (Illumina, San Diego, CA, USA) with paired-end 150 (PE150) read length [16]. The above process was completed by Shanghai Majorbio Biomedical Technology Co., Ltd. (Shanghai, China).

### 2.10. Sequencing Analysis

Filtering out low-quality data from raw data yielded high-quality data. The mapped reads were spliced and compared with the annotated information of the reference genome [17]. Gene expression levels were quantified using transcripts per million reads (TPM). Differentially expressed genes (DEGs) were identified using the DESeq2 software package (version 1.24.0), with screening criteria set at |log2fold change| ≥ 1 and *p* < 0.05 [18]. Gene Ontology (GO) functional annotation of differential genes was performed using Goatools (https://github.com/tanghaibao/Goatools, accessed on 1 February 2022). Kyoto Encyclopedia of Genes and Genomes (KEGG) pathway enrichment analysis of DEGs was performed using KOBAS (http://kobas.cbi.pku.edu.cn/home.do, accessed on 15 February 2022) [19].

### 2.11. Statistical Analyses

GraphPad Prism (version 8) was utilized to analyze the data and present the results as mean ± standard error of the mean (SEM). For the analysis of statistically significant differences, an unpaired two-tailed Student’s *t*-test was used; *p* < 0.05 was regarded as statistically significant.

## 3. Results

### 3.1. OP Supplementation Alleviated the Expansion of Mesangial Matrix, Renal Fibrosis, and Renal Inflammation

Our previous research results demonstrated that OP significantly reduced fasting blood glucose in *db*/*db* mice (339.5 mg/dL vs. 512.5 mg/dL). OP can improve glucose tolerance and reduce the homeostasis model assessment–insulin resistance index significantly (30.4 vs. 17.2) [13].

**Figure 2:**
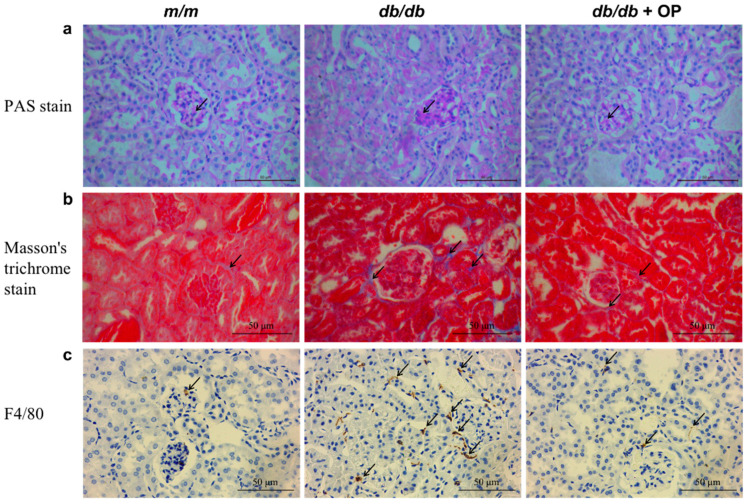
OP supplementation alleviated the expansion of the mesangial matrix, renal fibrosis, and renal inflammation. (**a**) PAS staining of the representative kidney (400× magnification), PAS staining positive area (arrows); scale bar = 50 μm; (**b**) Masson’s trichrome staining of the representative kidney (400× magnification), Masson’s trichrome staining positive area (arrows); (**c**) histo-immunostaining of the representative kidney (400× magnification), F4/80 positive area (arrows).

Diabetic nephropathy was characterized by expansion of the mesangial matrix, which eventually blocked the glomerular capillaries [10]. We found that the PAS-positive mesangial matrix areas (black arrows) were larger in *db*/*db* mice than in *m*/*m* mice. OP supplementation decreased the PAS-positive mesangial matrix areas (black arrows) (Figure 2a). By Masson staining, the *db*/*db* group showed increased renal fibrosis areas compared with the *m*/*m* group (black arrows). OP supplementation reduced the degree of renal fibrosis (Figure 2b). In addition, renal inflammation contributed to the progression of diabetic nephropathy [20]. F4/80 was a marker of macrophages. As shown in Figure 2c, the expression of F4/80 was higher in *db*/*db* mice compared with *m*/*m* mice (black arrows). In contrast, mice in the *db*/*db* + OP group showed fewer F4/80 positive areas than those in the *db*/*db* group.

### 3.2. OP Supplementation Inhibited Renal Apoptosis

**Figure 3:**
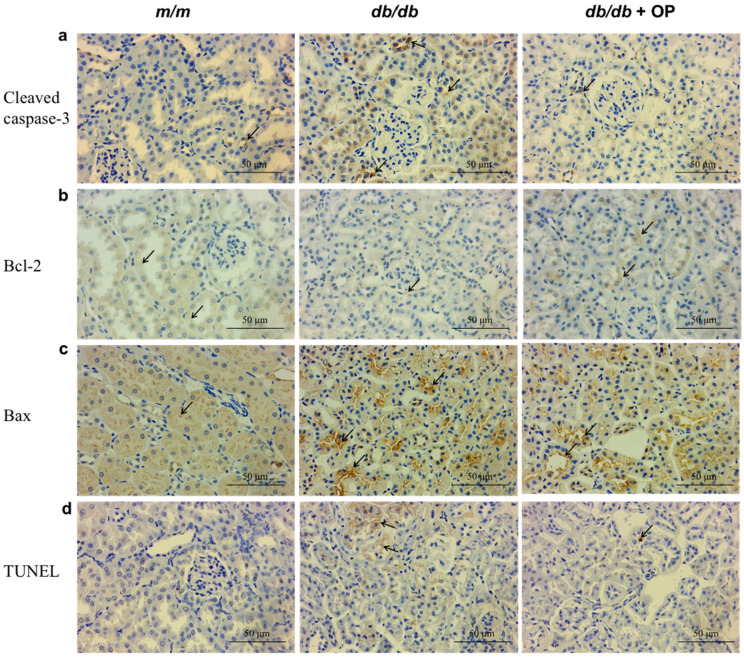
OP supplementation inhibited renal apoptosis. (**a**–**c**) Representative micrographs (400× magnification) of immunohistochemical pathological sections of cleaved caspase-3, Bcl-2, and Bax; (**d**) representative micrographs (400× magnification) of TUNEL-stained renal pathological sections. Arrows indicate positive staining regions.

Apoptosis is a factor that can lead to the onset and progression of diabetic nephropathy. Compared with the *m*/*m* group, the respective proportions of cleaved caspase-3 positive regions, Bax positive regions, and TUNEL staining positive regions in the *db*/*db* group were increased (Figure 3a,c,d) and the proportion of Bcl-2 positive regions was decreased (Figure 3b). However, these histopathology parameters were significantly rescued by OP supplementation. These results suggested that OP treatment can partially inhibit renal apoptosis.

### 3.3. Effects of OP on mRNA Levels of FIBROTIC Factors, Anti-Inflammatory and Pro-Inflammatory Factors, and Oxidase in the Kidney

**Figure 4:**
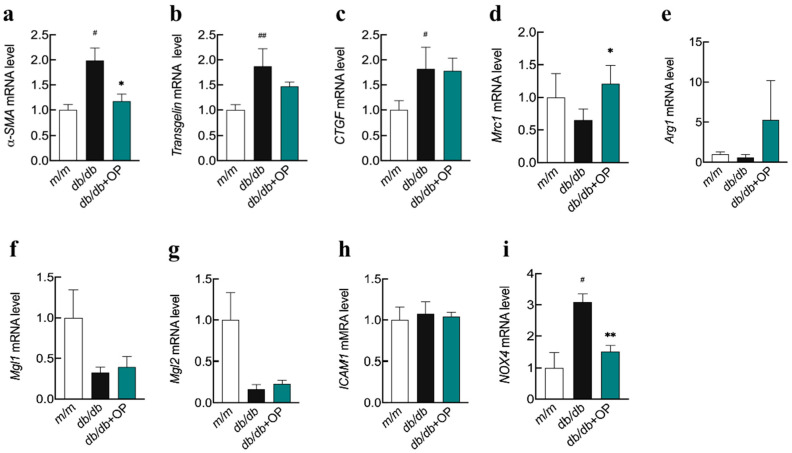
The mRNA levels of fibrotic factors, anti-inflammatory and pro-inflammatory factors, and oxidase. (**a**–**c**) mRNA expression levels of *a-SMA*, *Transgelin*, and *CTGF*; (**d**–**h**) mRNA expression levels of *Mrc1*, *Arg1*, *Mgl1*, *Mgl2*, and *ICAM1*; (**i**) mRNA expression levels of *NOX4*. Results were expressed as mean ± standard error (SEM) (*n* = 3). # *p* < 0.05, ## *p* < 0.01, *db*/*db* vs. *m*/*m*. * *p* < 0.05, ** *p* < 0.01, *db*/*db* + OP vs. *db*/*db*.

Furthermore, mRNA levels of fibrotic factors (α-smooth muscle actin (*α-SMA*), *transgelin*, and the connective tissue growth factor (*CTGF*)) were assessed. The results demonstrated that the mRNA levels of *α-SMA*, *transgelin*, and *CTGF* were significantly upregulated in *db*/*db* mice compared with *m*/*m* mice (Figure 4a–c). However, OP treatment significantly decreased the mRNA level of *α-SMA* (Figure 4a). Nevertheless, the comparisons between *db*/*db* and *db*/*db* + OP groups in relation to *transgelin* and *CTGF* showed no significant differences (Figure 4b,c). We then investigated the effects of OP on the mRNA levels of anti-inflammatory and pro-inflammatory factors. Compared with the *db*/*db* group, the mRNA level of the anti-inflammatory factor mannose receptor C type 1 (*Mrc1*) was significantly upregulated (Figure 4d) in the *db*/*db* + OP group. Dietary intake of OP had slight but not significant effects on the mRNA levels of the anti-inflammatory factors arginase 1 (*Arg1*), macrophage galactose-type lectin 1 (*Mgl1*), and macrophage galactose-type lectin 2 (*Mgl2*), as well as on the mRNA level of the pro-inflammatory factor intercellular adhesion molecule-1 (*ICAM1*) (Figure 4e–h). We also investigated the effect of OP on oxidase-related genes. Compared with the mice in the *m*/*m* group, the mRNA level of NADPH oxidases 4 (*NOX4*) in the *db*/*db* mice was significantly increased. However, OP treatment significantly decreased the mRNA level of *NOX4* (Figure 4i).

### 3.4. OP Supplementation Modifies Gene Expression Profiles of the Kidney

**Figure 5:**
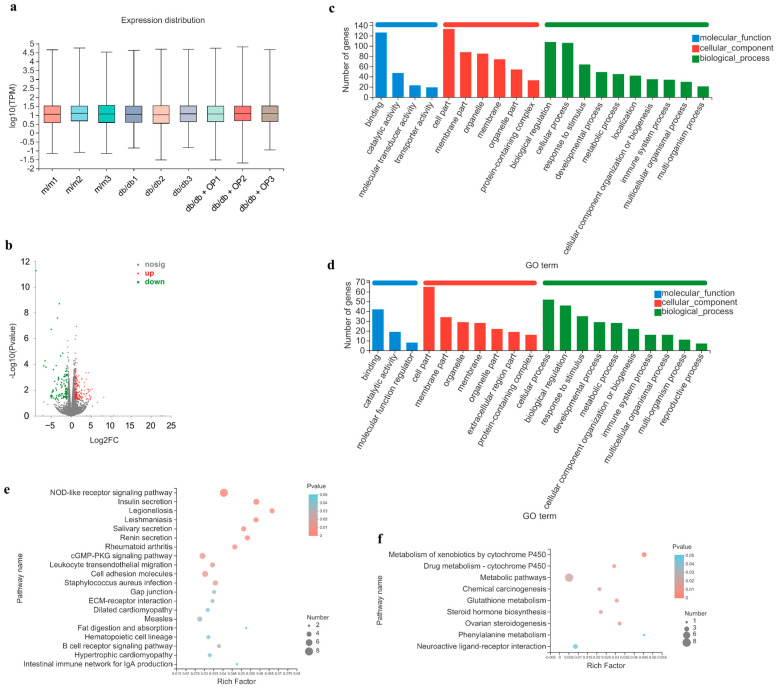
Changes in renal gene expression induced by OP in the mice. (**a**) Box plot indicating gene expression distribution for each sample; (**b**) volcano plot showing upregulated and downregulated genes induced by OP; (**c**,**d**) GO analysis of upregulated and downregulated DEGs in *db*/*db* + OP group compared with *db*/*db* group; (**e**,**f**) KEGG signaling pathway analysis of upregulated and downregulated DEGs in *db*/*db* + OP group compared with *db*/*db* group.

To investigate the potential underlying molecular mechanism of OP in alleviating long-course diabetic nephropathy, we conducted RNA-sequencing analysis of kidney tissues. Gene expression profiles were normalized using TPM (Figure 5a). A screening threshold of *p* < 0.05 and |log 2FC| ≥ 1 was applied to identify DEGs between the *db*/*db* and *db*/*db* + OP groups, as depicted in the volcano plot graph (Figure 5b). In total, we identified 290 DEGs, including 189 upregulated and 101 downregulated genes, between the *db*/*db* group and the *db*/*db* + OP group. These DEGs were analyzed using GO functional annotation and KEGG analysis. The GO terms with the top 20 enrichment degrees are shown in Figure 5c,d. According to the GO classification, the DEGs between the *db*/*db* and *db*/*db* + OP groups were involved in three categories, namely biological process, molecular function, and cellular component. Next, we performed KEGG pathway enrichment analysis (Figure 5e,f). By examining the top 20 key pathways (*p* < 0.05), we conducted an analysis to explore the specific pathways that differed between the *db*/*db* and *db*/*db* + OP groups in this study. Interestingly, some DEGs were notably enriched in certain pathways, including the cGMP–PKG signaling pathway, the ECM-receptor interaction signaling pathway, and the Gap junction signaling pathway, all of which have implications in diabetic nephropathy. The upregulation of cGMP–PKG and Gap junction signaling pathways, and the downregulation of the ECM receptor interaction signaling pathway, were considered to be beneficial to the improvement of diabetes nephropathy [21,22,23,24]. In our KEGG analysis results, OP upregulated the cGMP–PKG and ECM receptor interaction signaling pathways and downregulated the Gap junction signaling pathway. Considering the aforementioned results, we selected the cGMP–PKG signaling pathway as the focus for investigating the mechanism by which OP regulates long-course diabetic nephropathy.

### 3.5. OP Alleviated Cardiac Fibrosis and Inflammation

**Figure 6:**
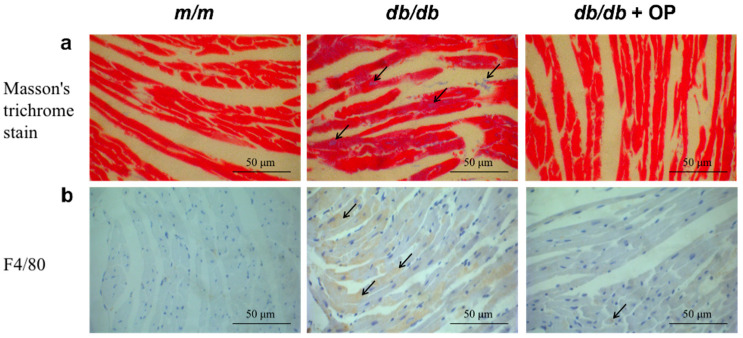
OP inhibited myocardial fibrosis and inflammation. (**a**) Representative micrographs of heart pathological sections stained by Masson’s trichrome (400×), Masson’s trichrome staining positive area (arrows); (**b**) representative micrographs of immunohistochemical pathological sections of F4/80 expression in the heart (400×), F4/80 positive area (arrows).

In our previous research, it was observed that mice in the *m*/*m* group exhibited neatly arranged myocardial fibers, whereas mice in the *db*/*db* group displayed disordered myocardial fibers. Following OP treatment, a notable improvement was observed, with more neatly arranged myocytes in the *db*/*db* + OP group [13]. We found that the cardiac fibrosis area in the *db*/*db* + OP group was smaller than that in the *db*/*db* group (Figure 6a). We also analyzed F4/80 expression in the heart tissue by immunohistochemical staining. Compared with that in the *db*/*db* group, the F4/80 expression level in the *db*/*db* + OP group was lower (Figure 6b). These results suggested that OP treatment ameliorated cardiac damage in *db*/*db* mice.

### 3.6. OP Supplementation Inhibited Cardiac Apoptosis

**Figure 7:**
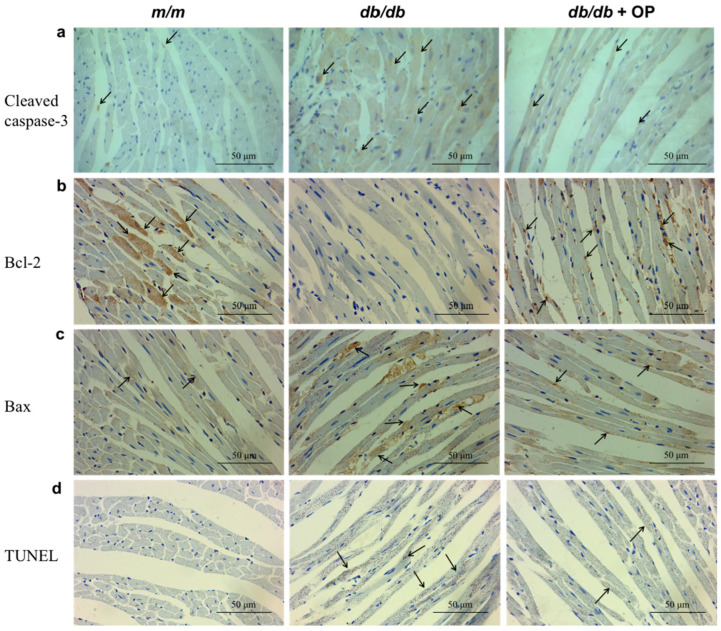
OP supplementation inhibited cardiac apoptosis. (**a**–**c**) Representative micrographs (400×) of immunohistochemical pathological sections of cleaved caspase-3, Bcl-2, and Bax expression in the heart; (**d**) representative micrographs (400×) of TUNEL-stained heart pathological sections. Arrows indicate positive staining regions.

Immunohistochemical staining and TUNEL staining were conducted to assess the level of cardiac apoptosis. Compared with that in the *db*/*db* group, the cleaved caspase-3 and Bax expression levels in the *db*/*db* + OP group were lower, whereas the Bcl-2 level was higher (Figure 7a–c). In addition, the results of the TUNEL staining were consistent with those of the immunohistochemical staining (Figure 7d). Therefore, our results suggested that OP supplementation can partially inhibit apoptosis in the heart of *db*/*db* mice.

### 3.7. OP Supplementation Modifies Gene Expression Profiles of the Heart

**Figure 8:**
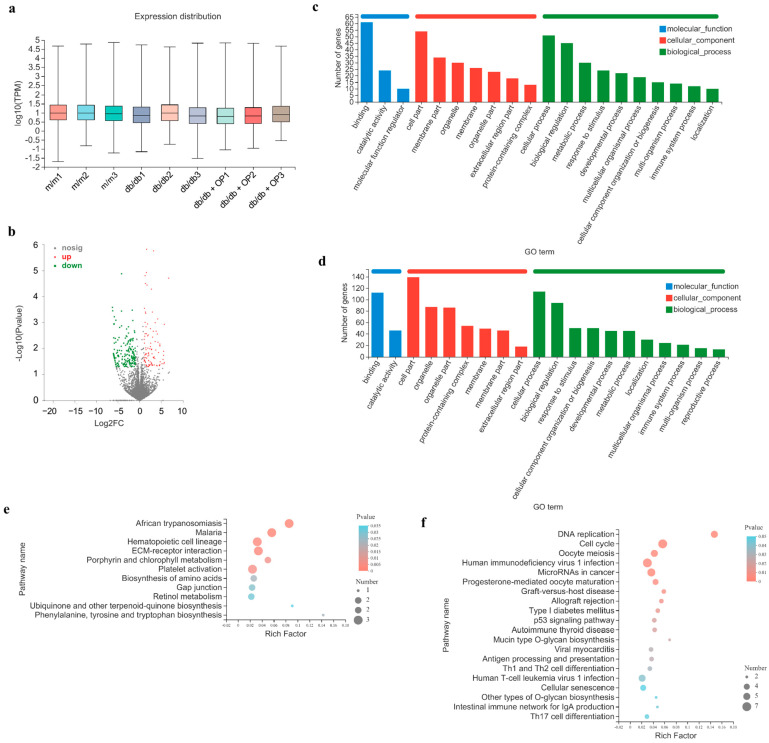
Changes in cardiac gene expression induced by OP in the mice. (**a**) Box plot indicating gene expression distribution for each sample; (**b**) volcano plot showing upregulated and downregulated genes induced by OP; (**c**,**d**) GO analysis of upregulated and downregulated DEGs in *db*/*db* + OP group compared with *db*/*db* group; (**e**,**f**) KEGG signaling pathway analysis of upregulated and downregulated DEGs in *db*/*db* + OP group compared with *db*/*db* group.

Next, we analyzed the gene expression profiles of heart tissues by RNA-sequencing, to verify the hypothesis that OP can alleviate long-course diabetic cardiomyopathy and to investigate the potential underlying molecular mechanism of the mitigation of long-course diabetic cardiomyopathy by OP. Gene expression distributions were normalized by TPM (Figure 8a). Figure 8b shows the scatter plot for the differential expression analysis of *db*/*db* vs. *db*/*db* + OP, including 267 DEGs, of which 96 were upregulated and 171 were downregulated. These DEGs were analyzed using GO functional annotation and KEGG analysis. The GO terms with the top 20 enrichment degrees are shown in Figure 8c,d. According to the GO classification, the DEGs between the *db*/*db* and *db*/*db* + OP groups were involved in three categories, namely biological process, molecular function, and cellular component. Next, we performed KEGG pathway enrichment analysis (Figure 8e,f). Based on the top 20 key pathways, we analyzed the specific pathways in the *db*/*db* and *db*/*db* + OP groups to gain insights into how OP may alleviate long-term diabetic cardiomyopathy. It was found that some DEGs were significantly enriched in the Gap junction signaling pathway, the p53 signaling pathway, and the cellular senescence signaling pathway, which are all associated with diabetic cardiomyopathy. We found that the role of OP on Gap junction signaling pathways was not clear. OP treatment downregulated p53 and cellular senescence signaling pathways. The downregulation of these signaling pathways was deemed beneficial for the amelioration of diabetic cardiomyopathy. Based on the above results, the p53 and cellular senescence signaling pathways were chosen to investigate the mechanism by which OP regulated long-course diabetic nephropathy.

### 3.8. qPCR Verification

**Figure 9:**
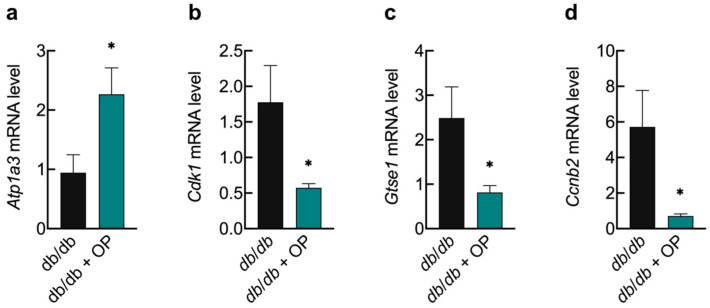
Validation of candidate DEGs using qPCR. (**a**) mRNA level of *Atp1a3* in the kidney; (**b**–**d**) mRNA levels of *Cdk1*, *Gtse1*, and *Ccnb2* in the heart. Results are expressed as mean ± standard error (SEM) (*n* = 3). * *p* < 0.05, *db*/*db* vs. *db*/*db* + OP.

In our research, 5 DEGs were involved in the cGMP–PKG signaling pathway, namely cGMP-dependent protein kinase 2 (*Prkg2*), potassium channel subfamily U member 1 (*Kcnu1*), ATPase Na^+^/K^+^ transporting subunit alpha 3 (*Atp1a3*), protein kinase cGMP-dependent 1 (*Prkg1*), and potassium calcium-activated channel subfamily M alpha 1 (*Kcnma1*). The RNA-sequencing data were consistent with the qPCR result, which confirmed that the mRNA level of *Atp1a3* was notably elevated in the kidneys of mice supplemented with OP (Figure 9a).

Three DEGs were involved in the p53 signaling pathway, namely cyclin-dependent kinase 1 (*Cdk1*), G two S phase expressed protein 1 (*Gtse1*), and cyclin B2 (*Ccnb2*). The cellular senescence signaling pathway involved 4 DEGs, namely *Cdk1*, *Ccnb2*, histocompatibility 2 T region locus 3 (*H2–T3*), and histocompatibility 2, Q region locus 2 (*H2–Q2*). Consistent with the RNA-sequencing data, the qPCR result validated the finding that *Cdk1*, *Gtse1*, and *Ccnb2* mRNA levels were significantly lower in the heart of mice supplemented with OP (Figure 9b–d).

## 4. Discussion

In the present study, we demonstrated the salutary effects of OP on long-course diabetic nephropathy and diabetic cardiomyopathy. Several studies have shown that OP exerts no lethality or toxic effects in animal experiments [25,26,27,28]. We also found no adverse effects during the whole experiment. The dosage of OP used in this study was 200 mg/kg. This dosage corresponded to a daily intake of approximately 16.2 mg/kg of human body weight (972 mg/60 kg), as calculated using the conversion method (body surface area normalization) suggested by Reagan-Shaw et al. [29]. People on a Mediterranean diet consume on average 20 olive fruits per day, which corresponds to a daily intake of about 25 mg OP [30]. The daily intake dose of 972 mg calculated in this paper is higher than 25 mg. The OP dosage used in this study corresponds to approximately 39 times the daily intake of OP in the Mediterranean diet. Foods containing OP that people are exposed to in their daily lives include olive oil, commercial olive leaf extracts, and others [31]. Since the content of OP is low in olive oil, commercial olive leaf extracts are good sources of OP supplementation [32]. The general content of OP in commercial olive leaf extracts ranges from 20% to 50%. Based on the 200 mg/kg dose used in this study, the conversion to a human (60 kg body weight) would be approximately 1 g/d. If the olive leaf extracts contain 50% OP, 2 g daily intake would be required.

Studies have shown that lowering the level of renal inflammation can reduce fibrosis and apoptosis, thereby alleviating diabetes nephropathy [33,34,35]. Renal inflammation can lead to the activation and recruitment of renal fibroblasts, start the fibrosis process, and trigger diabetes nephropathy [33,34,35]. Excessive apoptosis can induce renal injury and promote the development of diabetes nephropathy [10,36]. In the present study, the renal inflammation level was significantly decreased by OP supplementation (Figure 2c). Moreover, the results of the Masson staining pathology analysis confirmed that OP exerted a strong alleviating effect on kidney fibrosis, which was evidenced by the decreased *α-SMA* mRNA level (Figure 2b and Figure 4a). In addition, OP supplementation inhibited renal apoptosis (Figure 3). In agreement with previous research, OP showed favorable effects on decreasing apoptosis [10,37].

To gain further insights into the mechanism by which OP improves long-term diabetic nephropathy, we conducted transcriptomics and bioinformatics analyses. Transcriptome analysis suggested that OP may exert its beneficial effects by activating the cGMP–PKG signaling pathway. In this signaling pathway, PKG is activated by cGMP and transmits extracellular signals to the intracellular compartment. As an important second messenger, cGMP exerts regulatory effects on multiple physiological functions, such as decreasing apoptosis and inflammation [38,39]. In the kidney, medicines that increase the cellular content of cGMP may ameliorate chronic kidney disease [37,38,39,40,41]. A previous study showed that streptozotocin-induced reduction in glomerular cGMP production in diabetic rats triggers impaired renal endothelial diastolic function and disrupts normal renal function. Glomerular cGMP production was restored in diabetic rats after insulin treatment [42]. In our research, transcriptomic results showed that OP upregulated the cGMP–PKG signaling pathway in the kidney of *db*/*db* mice. We used qPCR to validate this hypothesis. Our results showed that the DEGs of *Atp1a3* were significantly increased when the cGMP–PKG signaling pathway was activated (Figure 9a). *Atp1a3* encodes a protein for the α3 subunit of Na^+^/K^+^–ATPase. Na^+^/K^+^–ATPase is located downstream of PKG and responsible for establishing and maintaining an electrochemical gradient of Na^+^ and K^+^ across the cytoplasmic membrane. Activation of ATP-dependent K^+^ channels exerted an ameliorative effect on diabetic nephropathy [43]. This suggested that the molecular mechanism by which OP ameliorated diabetic nephropathy in *db*/*db* mice may be related to upregulation of the cGMP–PKG signaling pathway.

In type 2 diabetes, chronic myocardium inflammation induces metabolic and cardiac structural changes, including myocardial fibrosis and cardiomyocyte apoptosis, which facilitate the diabetic heart failure phenotype [44]. In our research, the cardiac inflammation level was decreased by OP supplementation (Figure 6b). Furthermore, the results from the Masson staining pathology analysis confirmed that OP had a substantial effect in alleviating heart fibrosis, as illustrated in Figure 6a. In addition, OP supplementation inhibited cardiac apoptosis (Figure 7).

Similarly, in order to enhance our understanding of the regulatory mechanism by which OP improves long-term diabetic cardiomyopathy, transcriptomics and bioinformatics analyses were carried out. According to the transcriptome analysis, it is hypothesized that OP may ameliorate long-term diabetic cardiomyopathy by downregulating the p53 signaling pathway and the cellular senescence signaling pathway. The main functions of the p53 signaling pathway include inducing cell cycle arrest and promoting cell apoptosis [45,46,47]. p53 promotes apoptosis by activating caspase-3, as well as by upregulating the expression level of Bax and downregulating the expression of Bcl-2 [48,49]. Senescent cells are in a state of cell cycle arrest but maintain metabolic activity by secreting inflammatory factors. This secretory phenotype associated with senescence is a trigger for chronic inflammation [50]. Studies have shown that cellular senescence is associated with cardiovascular disease and may cause or exacerbate myocardial dysfunction in type 2 diabetes [51]. In the present study, OP downregulated the cellular senescence signaling pathway in the heart of *db*/*db* mice and ameliorated cardiac cell inflammation. Validation of three DEGs enriched in the signaling pathway of the p53 signaling pathway using qPCR revealed that the mRNA levels of *Cdk1*, *Gtse1*, and *Ccnb2* were significantly decreased in the OP group (Figure 9b–d). Validation of DEGs enriched in the cellular senescence signaling pathway revealed that OP significantly decreased the mRNA levels of *Cdk1* and *Ccnb2* in the heart of *db*/*db* mice (Figure 9b,d). These qPCR results were consistent with the transcriptomic results. The above results indicated that the synergy of multiple signaling pathways might directly or indirectly mediate the mitigation of OP supplementation on long-course diabetic cardiomyopathy.

Our transcriptome analysis results showed that the molecular mechanism by which OP alleviates long-course diabetes nephropathy was related to the upregulation of the cGMP–PKG signaling pathway, and that the molecular mechanism by which OP mitigates long-course diabetes cardiomyopathy was associated with the downregulation of the p53 and cellular senescence signaling pathways. Both upregulation of cGMP–PKG and downregulation of cell senescence signaling pathways can inhibit inflammation, which suggested that OP may improve long-course diabetes nephropathy and diabetes cardiomyopathy by inhibiting inflammation, though through different signaling pathways.

In the present study, we used transcriptome sequencing to explore the mechanism of OP treatment for these diabetes complications and validated the results at the mRNA level using qPCR. However, we did not further validate the results at the protein level. Therefore, western blot or proteomics methods can be used to excavate the mechanism of OP treatment for these diseases in the future.

## 5. Conclusions

In summary, the present research illustrates that OP supplementation alleviates long-term diabetic nephropathy and diabetic cardiomyopathy, as evidenced by the inhibition of inflammation, fibrosis, and apoptosis. The underlying mechanism of the mitigation effects of OP is associated with the upregulation of the cGMP–PKG signaling pathway in the kidney and the downregulation of the p53 and cellular senescence signaling pathways in the heart of *db*/*db* mice. These results indicate the potential of OP as a natural dietary functional ingredient to ameliorate long-course diabetic nephropathy and diabetic cardiomyopathy.

## Figures and Tables

**Table 1:** Primer sequences used for qPCR.

Genes	Gene Description	Forward Primer 5′-3′	Reverse Primer 5′-3′
*α-SMA*	α-smooth muscle actin	CCGCCATGTATGTGGCTATT	AGATAGGCACGTTGTGAGTC
*Transgelin*	transgelin	GCGACTAGTGGAGTGGATTG	GATCCCTCAGGATACAGGCT
*CTGF*	connective tissue growth factor	TGGCCCTGACCCAACTATGA	CTTAGAACAGGCGCTCCACTCT
*Mrc1*	mannose receptor C type 1	TGTGGTGAGCTGAAAGGTGA	CAGGTGTGGGCTCAGGTAGT
*Arg1*	arginase 1	GTATGACGTGAGAGACCACG	CTCGCAAGCCAATGTACACG
*Mgl1*	macrophage galactose-type lectin 1	ATGATGTCTGCCAGAGAACC	ATCACAGATTTCAGCAACCTTA
*Mgl2*	macrophage galactose-type lectin 2	TTAGCCAATGTGCTTAGCTGG	GGCCTCCAATTCTTGAAACCT
*Cyba*	cytochrome b light chain	GGAGCGATGTGGACAGAAGTA	GGTTTAGGCTCAATGGGAGTC
*Nrf2*	nuclear transcription factor erythroid 2-related factor 2	CCTAGGTCCTTGTTCCGCC	CTAGTCCGAGCAGCGGAGA
*ICAM1*	intercellular adhesion molecule-1	CAATGGCTTCAACCCGTGC	GTTCTCAAAGCACAGCGGAC
*NOX4*	NADPH oxidases 4	CCAAATGTTGGGCGATTGTGT	CAGGACTGTCCGGCACATAG
*Atp1a3*	ATPase Na^+^/K^+^ transporting subunit alpha 3	ACGGACCGACAGACGCAC	GCAGGATGGGCTCAGGC
*Cdk1*	cyclin-dependent kinase 1	AGAAGGTACTTACGGTGTGGT	GAGAGATTTCCCGAATTGCAGT
*Gtse1*	G two S phase expressed protein 1	AGAGGATCACCAGCAAGCTCCA	GTTTCGTCCTCTGAATGCTGGC
*Ccnb2*	cyclin B2	GCCAAGAGCCATGTGACTATC	CAGAGCTGGTACTTTGGTGTTC
*β-actin*	β-actin	GGCTGTATTCCCCTCCATCG	CCAGTTGGTAACAATGCCATGT

## Data Availability

Data is contained within the article.

## References

[1] Piccoli G.B., Grassi G., Cabiddu G., Nazha M., Roggero S., Capizzi I., De Pascale A., Priola A.M., Di Vico C., Maxia S. (2015). Diabetic kidney disease: A syndrome rather than a single disease. Rev. Diabet. Stud..

[2] Kanodia K.V., Vanikar A.V., Nigam L., Patel R.D., Suthar K.S., Patel H. (2017). Clinicopathological study of nondiabetic renal disease in type 2 diabetic patients: A single center experience from India. Saudi J. Kidney Dis. Transpl..

[3] Zhao Y.H., Li M.J., Wang Y.N., Geng R.X., Fang J.J., Liu Q., Kang S.K., Zeng W.C., Huang K.L., Tong T. (2023). Understanding the mechanism underlying the anti-diabetic effect of dietary component: A focus on gut microbiota. Crit. Rev. Food Sci. Nutr..

[4] Wang X., Kang J., Liu Q., Tong T., Quan H. (2020). Fighting diabetes mellitus: Pharmacological and non-pharmacological approaches. Curr. Pharm. Des..

[5] Wang Y.N., Liu Q., Kang S.K., Huang K.L., Tong T. (2021). Dietary bioactive ingredients modulating the cAMP signaling in diabetes treatment. Nutrients.

[6] Gupta S., Dominguez M., Golestaneh L. (2023). Diabetic kidney disease: An update. Med. Clin. N. Am..

[7] Murtaza G., Virk H.U.H., Khalid M., Lavie C.J., Ventura H., Mukherjee D., Ramu V., Bhogal S., Kumar G., Shanmugasundaram M. (2019). Diabetic cardiomyopathy—A comprehensive updated review. Prog. Cardiovasc. Dis..

[8] Zheng S.J., Huang K.L., Tong T. (2021). Efficacy and mechanisms of oleuropein in mitigating diabetes and diabetes complications. J. Agric. Food Chem..

[9] Ahamad J., Toufeeq I., Khan M.A., Ameen M.S.M., Anwer E.T., Uthirapathy S., Mir S.R., Ahmad J. (2019). Oleuropein: A natural antioxidant molecule in the treatment of metabolic syndrome. Phyther. Res..

[10] Liu Y., Dai W., Ye S. (2022). The olive constituent oleuropein exerts nephritic protective effects on diabetic nephropathy in *db*/*db* mice. Arch. Physiol. Biochem..

[11] Nekooeian A., Khalili A., Khosravi M. (2014). Oleuropein offers cardioprotection in rats with simultaneous type 2 diabetes and renal hypertension. Indian J. Pharmacol..

[12] Jemai H., Sayadi S. (2015). Heart histopathology and oxidative features in diabetic rats and protective effects of oleuropein. Adv. Biosci. Biotechnol..

[13] Zheng S.J., Wang Y.N., Fang J.J., Geng R.X., Li M.J., Zhao Y.H., Kang S.G., Huang K.L., Tong T. (2021). Oleuropein ameliorates advanced stage of type 2 diabetes in *db*/*db* mice by regulating gut microbiota. Nutrients.

[14] Kimura T., Kaneto H., Shimoda M., Hirukawa H., Okauchi S., Kohara K., Hamamoto S., Tawaramoto K., Hashiramoto M., Kaku K. (2015). Protective effects of pioglitazone and/or liraglutide on pancreatic β-cells in *db*/*db* mice: Comparison of their effects between in an early and advanced stage of diabetes. Mol. Cell. Endocrinol..

[15] Kimura T., Obata A., Shimoda M., Okauchi S., Kanda-Kimura Y., Nogami Y., Moriuchi S., Hirukawa H., Kohara K., Nakanishi S. (2018). Protective effects of the SGLT2 inhibitor luseogliflozin on pancreatic β-cells in *db*/*db* mice: The earlier and longer, the better. Diabetes Obes. Metab..

[16] Geng R.X., Fang J.J., Kang S.K., Huang K.L., Tong T. (2023). Chronic exposure to UVB induces nephritis and gut microbiota dysbiosis in mice based on the integration of renal transcriptome profiles and 16S rRNA sequencing data. Environ. Pollut..

[17] Kim D., Langmead B., Salzberg S.L. (2015). HISAT: A fast spliced aligner with low memory requirements. Nat. Methods.

[18] Love M.I., Huber W., Anders S. (2014). Moderated estimation of fold change and dispersion for RNA-seq data with DESeq2. Genome Biol..

[19] Xie C., Mao X., Huang J., Ding Y., Wu J., Dong S., Kong L., Gao G., Li C.Y., Wei L. (2011). KOBAS 2.0: A web server for annotation and identification of enriched pathways and diseases. Nucleic Acids Res..

[20] Jheng H.F., Hayashi K., Matsumura Y., Kawada T., Seno S., Matsuda H., Inoue K., Nomura W., Takahashi H., Goto T. (2020). Anti-inflammatory and antioxidative properties of isoflavones provide renal protective effects distinct from those of dietary soy proteins against diabetic nephropathy. Mol. Nutr. Food Res..

[21] Wang J., Zhang Q., Li S., Chen Z., Tan J., Yao J., Duan D. (2020). Low molecular weight fucoidan alleviates diabetic nephropathy by binding fibronectin and inhibiting ECM-receptor interaction in human renal mesangial cells. Int. J. Biol. Macromol..

[22] Yang F.Y., Cui Z.H., Deng H.J., Wang Y., Chen Y., Li H.Q., Yuan L. (2019). Identification of miRNAs-genes regulatory network in diabetic nephropathy based on bioinformatics analysis. Medicine.

[23] Takenaka T., Inoue T., Okada H., Ohno Y., Miyazaki T., Chaston D.J., Hill C.E., Suzuki H. (2011). Altered gap junctional communication and renal haemodynamics in Zucker fatty rat model of type 2 diabetes. Diabetologia.

[24] Chen Y., Burnett J.C. (2018). Particulate guanylyl cyclase A/cGMP signaling pathway in the kidney: Physiologic and therapeutic indications. Int. J. Mol. Sci..

[25] Petkov V., Manolov P. (1972). Pharmacological analysis of the iridoid oleuropein. Arzneimittelforschung.

[26] Alonso J. (2004). Tratado de Fitofármacos y Nutracéuticos (Treaty of Phytopharmaceuticals and Nutraceuticals).

[27] Soliman G.A., Saeedan A.S., Abdel-Rahman R.F., Ogaly H.A., Abd-Elsalam R.M., Abdel-Kader M.S. (2019). Olive leaves extract attenuates type II diabetes mellitus-induced testicular damage in rats: Molecular and biochemical study. Saudi Pharm. J..

[28] Clewell A.E., Béres E., Vértesi A., Glávits R., Hirka G., Endres J.R., Murbach T.S., Szakonyiné I.P. (2016). A Comprehensive Toxicological Safety Assessment of an Extract of *Olea Europaea L.* Leaves (Bonolive™). Int. J. Toxicol..

[29] Reagan-Shaw S., Nihal M., Ahmad N. (2008). Dose translation from animal to human studies revisited. FASEB J..

[30] Zoidou E., Melliou E., Gikas E., Tsarbopoulos A., Magiatis P., Skaltsounis A.L. (2010). Identification of Throuba Thassos, a traditional Greek table olive variety, as a nutritional rich source of oleuropein. J. Agric. Food Chem..

[31] Porto A.D., Brosol G., Casarsa V., Bulfone L., Scandolin L., Catena C., Sechi L.A. (2022). The pivotal role of oleuropein in the anti-diabetic action of the mediterranean diet: A concise review. Pharmaceutics.

[32] Susalit E., Agus N., Effendi I., Tjandrawinata R.R., Nofiarny D., Perrinjaquet-Moccetti T., Verbruggen M. (2011). Olive (*Olea europaea*) leaf extract effective in patients with stage-1 hypertension: Comparison with Captopril. Phytomedicine.

[33] Kanasaki K., Taduri G., Koya D. (2013). Diabetic nephropathy: The role of inflammation in fibroblast activation and kidney fibrosis. Front. Endocrinol..

[34] Navarro-Gonzalez J.F., Mora-Fernandez C. (2008). The role of inflammatory cytokines in diabetic nephropathy. J. Am. Soc. Nephrol..

[35] Lim A.K.H., Tesch G.H. (2012). Inflammation in Diabetic Nephropathy. Mediators Inflamm..

[36] Sanchez-Nino M.D., Sanz A.B., Lorz C., Gnirke A., Rastaldi M.P., Nair V., Egido J., Ruiz-Ortega M., Kretzler M., Ortiz A. (2010). BASP1 promotes apoptosis in diabetic nephropathy. J. Am. Soc. Nephrol..

[37] Karabag F., Hazman O., Bozkurt M., Ince S. (2017). Antioxidant status and anti-inflammatory effects of oleuropein in streptozotocin-induced diabetic nephropathy in rats. European J. Med. Plants.

[38] Schlossmann J., Schinner E. (2012). cGMP becomes a drug target. Naunyn Schmiedeberg’s Arch. Pharmacol..

[39] Shen K., Johnson D.W., Gobe G.C. (2016). The role of cGMP and its signaling pathways in kidney disease. Am. J. Physiol. Renal Physiol..

[40] Burnett J.C., Buglioni A. (2015). New pharmacological strategies to increase cGMP. Annu. Rev. Med..

[41] Schinner E., Wetzl V., Schlossmann J. (2015). Cyclic nucleotide signalling in kidney fibrosis. Int. J. Mol. Sci..

[42] Wang Y.X., Brooks D.P., Edwards R.M. (1993). Attenuated glomerular cGMP production and renal vasodilation in streptozotocin-induced diabetic rats. Am. J. Physiol.-Regul. Integr. Comp. Physiol..

[43] Tanabe K., Lanaspa M.A., Kitagawa W., Rivard C.J., Miyazaki M., Klawitter J., Schreiner G.F., Saleem M.A., Mathieson P.W., Makino H. (2012). Nicorandil as a novel therapy for advanced diabetic nephropathy in the eNOS-deficient mouse. Am. J. Physiol. Renal Physiol..

[44] Ramesh P., Yeo J.L., Brady E.M., McCann G.P. (2022). Role of inflammation in diabetic cardiomyopathy. Ther. Adv. Endocrinol. Metab..

[45] Rufini A., Tucci P., Celardo I., Melino G. (2013). Senescence and aging: The critical roles of p53. Oncogene.

[46] Bensaad K., Vousden K.H. (2007). p53: New roles in metabolism. Trends Cell Biol..

[47] Lowe S.W., Cepero E., Evan G. (2004). Intrinsic tumour suppression. Nature.

[48] Sha J., Sui B., Su X., Meng Q., Zhang C. (2017). Alteration of oxidative stress and inflammatory cytokines induces apoptosis in diabetic nephropathy. Mol. Med. Rep..

[49] Miyashita T., Krajewski S., Krajewska M., Wang H.G., Lin H.K., Liebermann D.A., Hoffman B., Reed J.C. (1994). Tumor suppressor p53 is a regulator of bcl-2 and bax gene expression in vitro and in vivo. Oncogene.

[50] Shakeri H., Lemmens K., Gevaert A.B., De Meyer G.R.Y., Segers V.F.M. (2018). Cellular senescence links aging and diabetes in cardiovascular disease. Am. J. Physiol. -Hear. Circ. Physiol..

[51] Henson S.M., Aksentijevic D. (2021). Senescence and type 2 diabetic cardiomyopathy: How young can you die of old age?. Front. Pharmacol..

